# Bilateral inflammatory recurrence of HER-2 positive breast cancer: a unique case report and literature review

**DOI:** 10.3389/fonc.2024.1276637

**Published:** 2024-01-12

**Authors:** Rong Qin, Xiangyang Wang, Tingting Fan, Ting Wu, Chao Lu, Xun Shao, Liang Yin

**Affiliations:** ^1^ Department of Medical Oncology, Jiangsu University Affiliated People’s Hospital, Zhenjiang Clinical Medical College of Nanjing Medical University, Zhenjiang, China; ^2^ Department of Traditional Chinese Medicine, Jiangsu University Affiliated People’s Hospital, Clinical Medical College, Nanjing University of Chinese Medicine, Zhenjiang, China; ^3^ Department of Pathology, Jiangsu University Affiliated People’s Hospital, Zhenjiang, China; ^4^ Department of Medical Iconography, Jiangsu University Affiliated People’s Hospital, Zhenjiang, China; ^5^ Department of Nuclear Medicine, Jiangsu University Affiliated People’s Hospital, Zhenjiang, China; ^6^ Department of Breast Surgery, Jiangsu University Affiliated People’s Hospital, Zhenjiang, China

**Keywords:** inflammatory breast cancer, bilateral recurrence, HER-2 positive breast cancer, case report, whole-exome sequencing

## Abstract

Inflammatory breast cancer (IBC) is an aggressive and rare form of breast cancer with a poor prognosis. The occurrence of bilateral IBC in a short period of time is extremely rare. In this case report, a 54-year-old woman diagnosed with invasive ductal carcinoma of the left breast underwent lumpectomy, lymph node dissection, chemotherapy, and radiotherapy but opted against trastuzumab treatment. Four years later, she experienced bilateral breast inflammation, skin changes, edema, and heat (calor). Biopsies confirmed breast cancer metastasis to both breasts. Whole-Exome Sequencing revealed genetic mutations, including PIK3CA and C4orf54, in both primary and recurrent tumors, with significant downregulation in the recurrent tumors. KEGG analysis suggested potential enrichment of axon guidance signal pathways in both tumors. The patient showed a partial response after treatment with liposome paclitaxel, along with targeted therapy using trastuzumab and pertuzumab. This case report sheds light on the rare occurrence of bilateral inflammatory breast cancer post-HER-2 treatment and highlights the importance of genetic profiling in understanding the disease. Further research on clinical targets for breast cancer management is warranted.

## Introduction

Inflammatory breast cancer (IBC) represents a rare and highly aggressive type of invasive breast cancer, constituting just 2.5% of all breast cancer cases. Historically, its prognosis has been notably poor (1, 2). Among all molecular subtypes, triple-negative IBC patients experience the worst prognosis, with a 10-year overall survival rate of merely 17.8% (3). Typical clinical characteristics of IBC include involvement of ≥30% of the affected breast and/or skin, along with erythema, skin changes like peau d’orange, nipple inversion, edema, and warmth, often without an underlying palpable mass (2). Although IBC cells exhibit histopathological similarities with non-IBC breast cancer cells, they are usually distributed in clusters throughout the breast and skin, leading to common false negative imaging results. To distinguish IBC from non-IBC, pathological confirmation of invasive carcinoma is essential. When a patient presents with a strong suspicion of IBC on the basis of medical history and clinical signs, it is highly recommended to conduct breast imaging (e.g., mammography or ultrasound) and perform a tissue biopsy for definitive pathological confirmation (1, 4).

Breast cancer is classified into different molecular subtypes, namely hormone receptor (HR)-positive (defined by estrogen receptor-positive and/or progesterone receptor-positive), HER2-positive (also known as ERBB2), and triple-negative breast cancer (TNBC) (5). These subtypes are present not only in non-IBC but also in IBC, albeit with varying proportions (6). The occurrence of HER2-positive and triple-negative breast cancer is more frequent in IBC cases than in non-IBC cases. HER2-positive breast cancer accounts for up to 50% of IBC cases, while in non-IBC, it constitutes 20-25% of cases. Similarly, TNBC accounts for 10-15% of IBC cases, compared to 30% in non-IBC (7, 8). Therefore, HER2-positive breast cancer exhibits the highest incidence among IBC cases. As a result of these more aggressive phenotypes, distant metastasis often arises, impacting various locations such as the bone, lung, and liver. Patients with HER2-positive IBC frequently encounter relapse in the central nervous system (CNS) as their initial site of recurrence (9). It is worth mentioning that there is a scarcity of literature documenting instances of bilateral recurrence of IBC in patients with HER2-positive breast cancer.

## Case report

**Figure 1:**
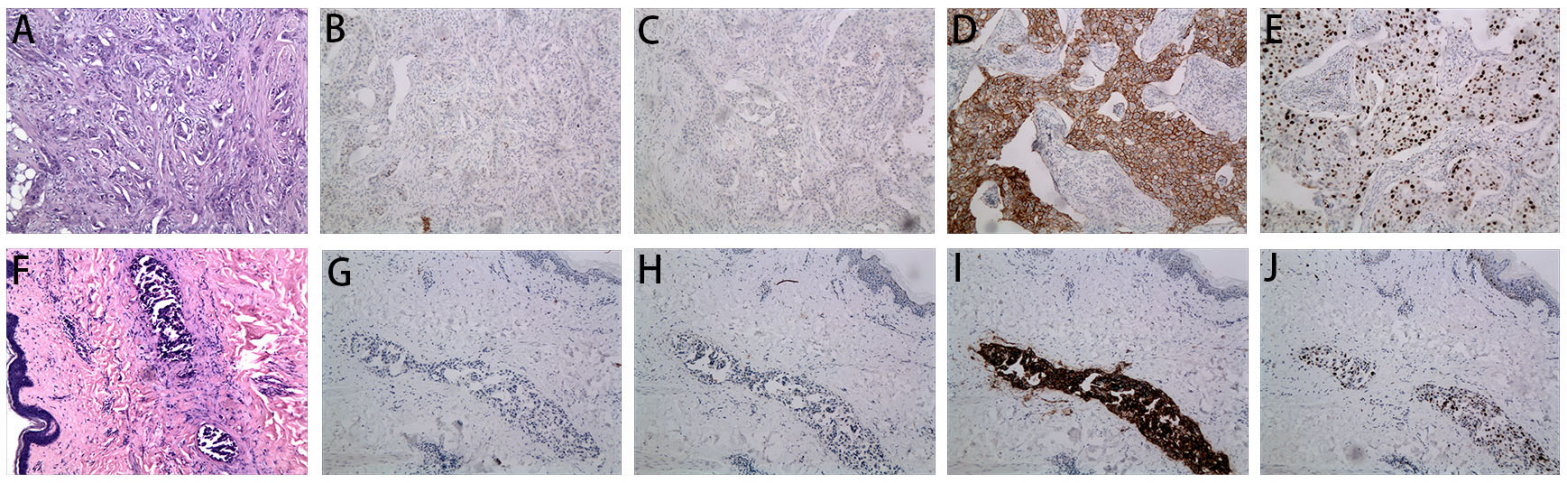
Microscopy examination of the breast specimen. **(A)** Postoperative HE staining suggested invasive ductal carcinoma of breast in 2018 (original magnification, 100 ×); The immunohistochemical staining of ER (negative, **B**), PR (negative, **C**), c-erbB2 (positive, **D**) and Ki 67 (40% positive, **E**) in 2018 (100 ×); **(F)** HE staining of the skin biopsy from the recurrent inflammation site showed lymphovascular tumor emboli and lymphatic dilatation in the superficial dermis. (original magnification, 100 ×); The immunohistochemical staining of skin biopsy at the site of inflammatory recurrence of ER (negative, **G**), PR (negative, **H**), c-erbB2 (positive, **I**) and Ki 67 (30% positive, **J**) in 2022 (original magnification, 100 ×).

A 58-year-old woman with a history of left-sided breast cancer (BC) diagnosed at age 54 in May 2018 underwent left-breast lumpectomy and axillary lymph node dissection. The postoperative pathology confirmed a grade 2 infiltrating ductal carcinoma (**Figure 1A**). Lymph nodes analysis showed no signs of metastasis, resulting in a TNM staging of pT2N0M0, Stage IIA. Immunohistochemical staining indicated negative results for estrogen receptor (ER) and progesterone receptor (PR), while being positive for human epidermal growth factor receptor 2 (c-erbB2) and Ki 67 (40%+) (**Figures 1B–E**). From May 2018 to October 2018, the patient received adjuvant chemotherapy with four cycles of Epirubicin and Cyclophosphamide followed by four cycles of docetaxel. However, for economic reasons, she declined targeted anti-HER-2 treatment with trastuzumab. Subsequently, she underwent postoperative adjuvant local radiotherapy, with a total dose of 50 Gy at 2 Gy/day × 25 fractions to the whole left breast, completing the radiotherapy in January 2019. Following treatment, the patient was under surveillance with clinic follow-ups every 3-6 months, focusing on tumor biomarkers and imaging monitoring in the local hospital. Notably, there had been no evidence of loco-regional or distant recurrence.

**Figure 2:**
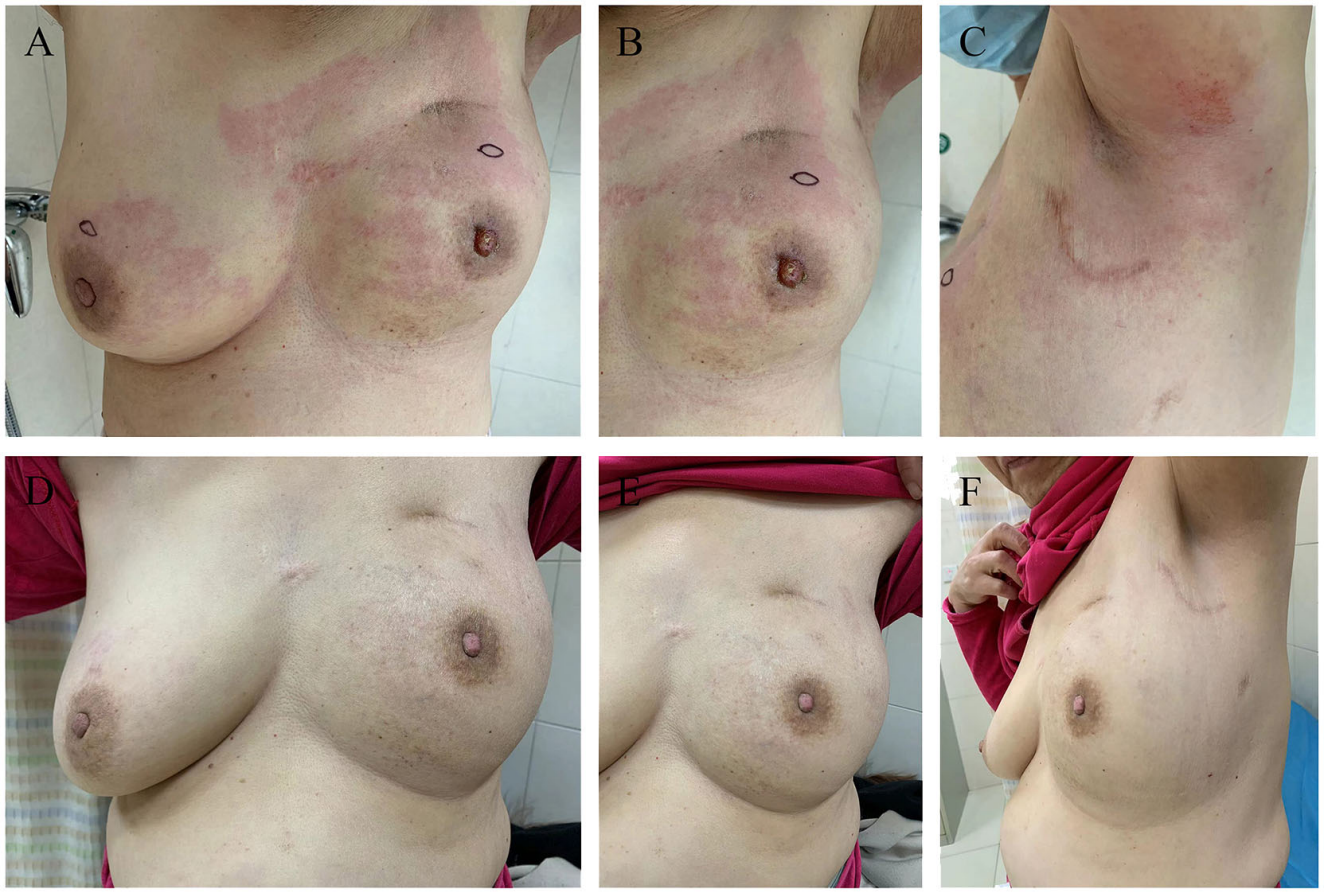
Photographic evidence showing the improvement of the disease (all provided by the patient for literature and education). **(A-C)** indicates the skin presented with erythema, peau d’orange, inflamed and oedematous of the bilateral breast and a nipple retraction of left breast (taken in September 2022). **(D-F)** indicates significant improvement in the presentation of skin with inflammatory recurrence after six cycles of chemotherapy and targeted therapy (taken in December 2022).

**Figure 3:**
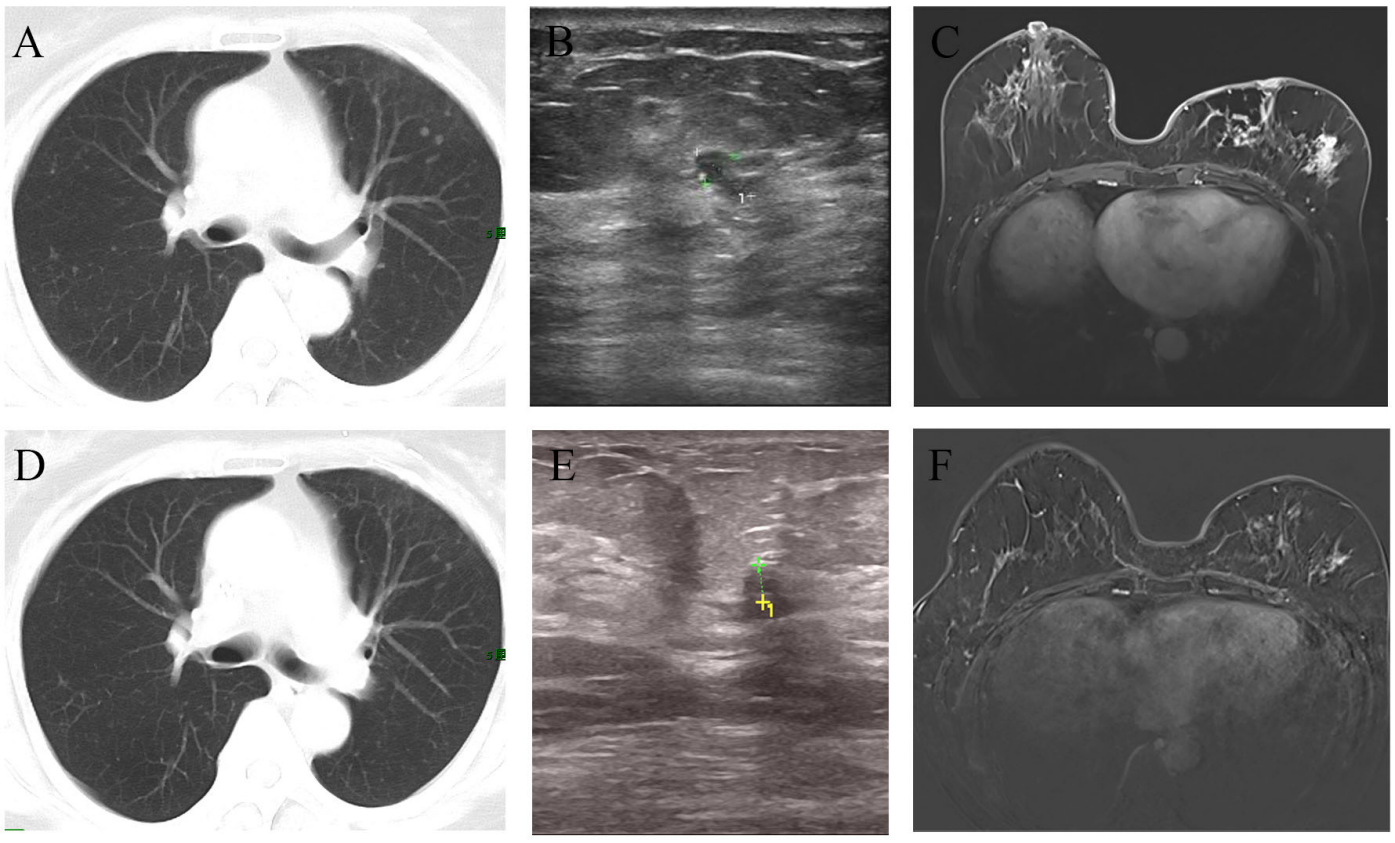
Imaging changes before and after treatment. The CT scan suggested multiple metastases in both lungs **(A)**. After chemotherapy combined with targeted therapy, the pulmonary metastatic lesions have significantly decreased compared to before **(D)**. Ultrasound revealed an irregular hypoechoic area measuring approximately 7*4mm adjacent to the left nipple in the 3 o’clock direction of the breast, with indistinct borders **(B)**. After treatment, the ultrasound indicated that the hypoechoic area has reduced in size to approximately 3.5*3.3mm compared to before **(E)**. MRI showed the presence of an irregular ring-enhancing lesion measuring approximately 11*11mm in the central region of the left breast. Additionally, there is thickening of the skin overlying the left breast with thickened skin **(C)**. After treatment, MRI indicated the absence of the previously visible nodule in the central region of the left breast **(F)**.

In September 2022, the patient presented at the hospital with vague breast pain. Physical examination revealed erythema, peau d’orange with skin thickening, inflammation, and edema of both breasts, along with left breast nipple retraction, and no palpable axillary nodes (**Figures 2A–C**). A chest computed tomography (CT) scan showed thickening skin of both breasts, multiple small nodules in the left breast, and multiple lung metastases (**Figure 3A**). Ultrasound scanning detected a suspicious node in her left breast (**Figure 3B**). Magnetic resonance imaging (MRI) of the breasts indicated the presence of a node in the center and upper external region of the left breast with BI-RADs:4a-4c (**Figure 3C**). A biopsy of the inflamed skin in both breasts confirmed breast cancer metastasis (**Figure 1F**). Immunohistochemistry results showed negative estrogen receptor (ER) and progesterone receptor (PR) status, c-erbB2 2+ expression, and positive Ki 67 (30%) (**Figures 1G–J**). Additionally, the fluorescence *in situ* hybridization (FISH) test confirmed c-erbB2 amplification.

**Figure 4:**
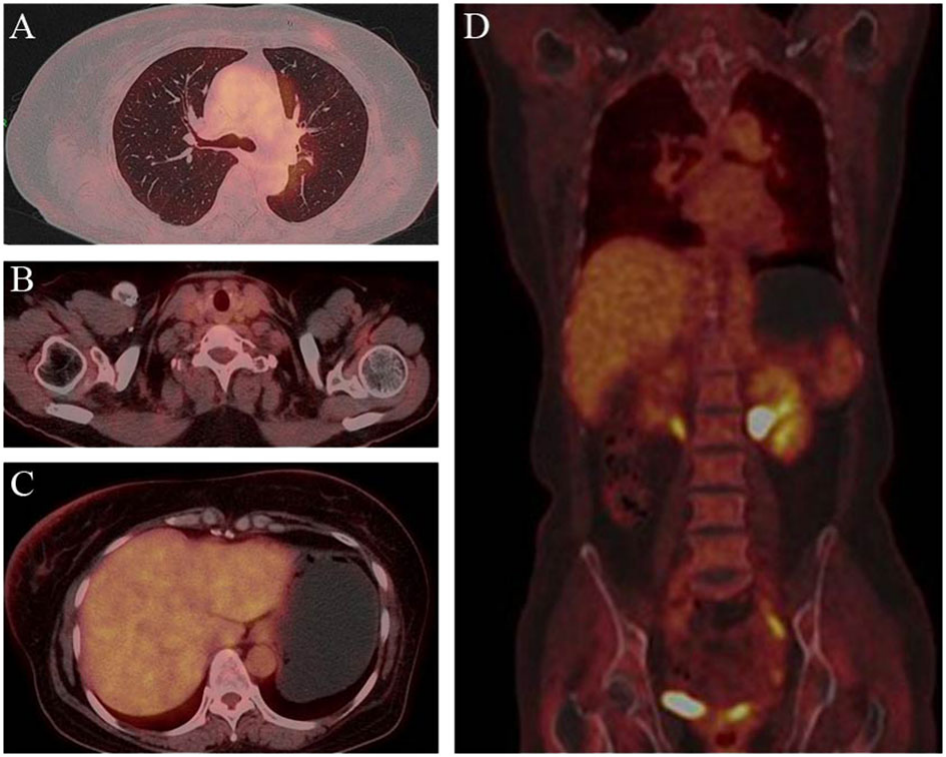
Post-treatment PET/CT findings. PET demonstrated clearcut accumulation of 18F fluorodeoxyglucose in lung **(A)**, lymph nodes in the clavicular region **(B)**, liver **(C)** and whole body **(D)**.

Further metastatic surveys, including CT scans of the abdomen, pelvis, brain, and whole-body bone scanning, yielded negative results. Blood analysis and tumor markers like carbohydrate antigen 153 (CA 15-3) were within normal ranges. A multi-disciplinary review led to the decision for rescue treatment of advanced breast cancer. The patient received six cycles of liposome paclitaxel and targeted therapy with trastuzumab (initially 8 mg/kg followed by 6 mg/kg every 3 weeks) and pertuzumab (initially 840mg followed by 420mg every 3 weeks). During treatment, inflammation and erythema of the breast skin gradually decreased, and bilateral lung metastases showed improvement (**Figures 2D–F**). Follow-up CT, ultrasound, and MRI scans following liposome paclitaxel demonstrated excellent response (**Figures 3D–F**). At the end of treatment, positron emission tomography/computed tomography (PET/CT) did not show pulmonary or other visceral organ metastasis (**Figure 4**). The patient continued targeted treatment with trastuzumab and pertuzumab every 3 weeks, along with oral capecitabine for maintenance chemotherapy.

In this study, we present the diagnosis and treatment of a patient with inflammatory recurrence of breast cancer. To further investigate genetic alterations, particularly variants of unknown significance (VUS), we employed Whole Exome Sequencing (WES). The primary tumor (P) and skin recurrence (R) of the patient exhibited a Tumor Mutation Burden (TMB) of 0.12 and 0.81 mut/Mb, respectively. Through WES analysis (10), a total of 111 gene variants were identified in the primary tumor (P) and 28 gene variants in the recurrent tumor (R). Interestingly, two variants (PIK3CA and C4orf54) were found to be present in both samples. The abundance of PIK3CA was found to be 22.22% in the primary tumor (P) and 2.99% in the recurrent tumor (R), while the abundance of C4orf54 was 30.95% in P and 4.01% in R (**Table 1**). KEGG analysis revealed potential enrichment of axon guidance signal pathways in both the primary tumor (P) and recurrent tumor (R) (**Supplementary Figure S1**). Molecular function analysis indicated that P may show changes in the activity of G protein-coupled neurotransmitter receptors, while R may exhibit changes in the activity of PI3 kinase (**Supplementary Figure S2**). Moreover, cellular component analysis revealed the detection of changes in the semaphorin receptor complex in both P and R (**Supplementary Figure S3**). These findings shed light on the genetic alterations and potential signaling pathways involved in the recurrence of inflammatory breast cancer in this patient.

**Table 1:** WES was used to analyze gene point mutations, deletions, insertions, and other genetic alterations in both the primary tumor (P) and skin recurrence (R) in breast cancer.

Gene	Mutant	Abundance(P)	Abundance(R)
PIK3CA	c.3140A>G (p.H1047R)	22.22%	2.99%
TP53	c.991C>T(p.Q331*)	–	3.57%
ASB11	c.623A>G (p.D208G)	9.23%	2.59%
C4orf54	c.4966G>T (p.A1656S)	30.95%	4.01%
CMYA5	c.2611C>T (p.P871S)	–	3.94%
EIF4G1	c.1428_1429del (p.S476Rfs*43)	–	3.27%
GOT1	c.277A>G (p.S93G)	–	2.34%
GPR156	c.852G>A (p.W284*)	–	2.77%
HIST1H4K	c.128G>A (p.G43D)	–	3.68%
HTR1F	c.470C>G (p.P157R)	–	2.39%
HYI	c.272A>C (p.E91A)	–	3.49%
KIF1A	c.3442C>G (p.L1148V)	–	2.38%
KLHL33	c.89T>C (p.F30S)	–	3.56%
KRT19	c.905_912delinsA(p.T302Kfs*11)	–	3.29%
LRP1	c.7370G>A (p.R2457H)	–	2.93%
MCC	c.578C>A (p.P193R)	–	3.04%
MUC16	c.5621G>A (p.R1874K)	–	2.88%
NAT10	c.2781G>T (p.E927D)	–	2.74%
PLEKHG2	c.146C>T (p.S49F)	–	2.19%
PLXNA3	c.4718T>G (p.V1573G)	–	4.27%
PLXNB3	c.1256C>T (p.P419L)	23.08%	3.07%
POLE2	c.852T>G (p.D284E)	–	2.69%
PTPRJ	c.2954T>C (p.L985P)	–	4.69%
RBMXL3	c.734C>T (p.P245L)	–	2.83%
TXNDC17	c.206G>A (p.C69Y)	–	2.50%
YTHDF1	c.1495A>C (p.N499H)	–	2.27%
ZSCAN12	c.452G>A (p.R151H)	–	3.38%

(*Tumor-specific mutations).

## Discussion

Only few cases of bilateral IBC have been reported in the past. One case report describes a patient with contralateral recurrence of IBC less than a year after the initial diagnosis (11). In the context of IBC, the incidence rate of HER2 positive breast cancer is as high as 50%, whereas in non-IBC, it ranges from 20-25%. Over the past decade, there have been significant advancements in improving the overall survival (OS) of HER2 positive metastatic breast cancer (MBC). However, despite these improvements, the survival rate for *de novo* MBC remains considerably higher than that of recurrent disease (12).

At present, there are many anti-HER2 therapies approved by the Food and Drug Administration (FDA), mainly including monoclonal antibodies (MAbs), the most representative of which are trastuzumab and pertuzumab, and small-molecule tyrosine kinase inhibitors (TKIs), such as lapatinib, tukatinib, and neratinib (13, 14). In recent years, antibody drug conjugates (ADC), such as ado-trastuzumab emtansine (T-DM1) and trastuzumab deruxtecan (T-DXd), also have good effects in HER2 positive patients with advanced breast cancer (15, 16). These different types of anti-HER2 drugs target anti-HER2 treatment through different mechanisms. According to the guidelines, THP regimen (paclitaxel, trastuzumab and pertuzumab) is recommended as the first-line treatment regimen for patients with HER-2 positive advanced breast cancer who were previously sensitive to trastuzumab treatment. The large, randomized, phase III CLEOPATRA clinical trial indicated that the median OS was 56.5 months in the group receiving the pertuzumab, trastuzumab and docetaxel, as compared with 40.8 months in the group receiving the trastuzumab, docetaxel and placebo. The study established the standard of care for treatment of patients with HER2‐positive breast cancer in the front‐line setting (17). In a Korean real-world study, the clinical outcomes of metastatic HER2-positive breast cancer patients treated with THP regime further proved the authenticity of the CLEOPATRA trial (18). Similarly, the study of weekly paclitaxel combined with trastuzumab and pertuzumab showed a longer follow-up of nearly 5 years and the median PFS was 24.2 months whereas the median OS was not reached for the overall group (19). Despite the fact that weekly paclitaxel has been demonstrated to have better tolerance compared to every-3-week docetaxel, a larger number of patients opt for the three-week regimen due to its convenience in daily life (20).

IBC and non-IBC are mainly differentiated by their clinical manifestations, but pathological confirmation is also essential to confirm the diagnosis of invasive cancer, which involves numerous dermal tumor emboli in the dermis overlying the breast (21). Among the four molecular types of breast cancer, the most prominent distinction between IBC and non-IBC lies in the overexpression of HER2-positive and triple-negative subtypes (22). Long-term survival rates for IBC patients are reported to be around 40%, and even with targeted treatment for HER2-positive IBC patients, resistance to HER2 targeted therapy often develops within two years, highlighting the urgency to explore disease pathogenesis and provide more treatment options (23, 24). Notably, IBC cases typically lack a single dominant tumor mass. Instead, tumor cells infiltrate loosely in the form of cell groups, clustering in both the matrix and lymphatic vessels (25). To identify potential therapeutic targets, Ross et al. employed next-generation sequencing technology to study metastatic parts of 53 IBC patients. The study identified TP53 and MYC as the most frequently altered genes, along with components of signaling pathways such as the RAS and phosphoinositide 3-kinase (PI3K) pathways. Within the RAS pathway, mutations were predominantly found in genes encoding ERBB2, KRAS, BRAF, and EGFR, while the PI3K pathway exhibited mutations in PIK3CA, PTEN, AKT1, and AKT3 (26). These findings provide crucial insights into potential avenues for targeted therapy in IBC.

In this patient, we adopted the classic three-week THP regime. Following the first cycle of chemotherapy and targeted therapy, the skin manifestations of metastatic inflammatory breast cancer showed progressive improvement without any safety issues. After completing the entire chemotherapy cycle, a comprehensive PET/CT evaluation revealed increased FDG uptake in the bilateral nipples and surrounding skin, with multiple small lung nodules significantly improved compared to before. Based on RECIST 1.1 criteria, the patient achieved partial response (PR). Moving forward, we are now considering the next step of treatment, which involves choosing between targeted maintenance therapy alone or combining targeted therapy with other drugs. Capecitabine, a well-established oral chemotherapy drug, has demonstrated positive maintenance treatment effects in numerous studies when combined with other therapies (27–29). For the follow-up maintenance treatment, we decided to use capecitabine in combination with trastuzumab and pertuzumab. This approach aims to provide the patient with the most effective and personalized treatment to further manage the disease.

At present, the patient’s condition remains stable and well-tolerated. Given the frequent relapse and distant metastasis observed in HER-2 positive breast cancer, we conducted WES to further investigate gene and pathway changes, aiming to understand the mechanisms driving metastasis and lay the groundwork for future treatments. We found alterations in G protein-coupled receptor activity (molecular function) in pre-treatment inflammatory breast cancer (IBC) tumors. Cellular component analysis revealed the presence of the “semaphorin receptor complex.” These findings align with Zare et al’s research, identifying G protein-coupled receptors as the primary pathway in pre-treatment IBC samples. Notably, the Semaphorin-3E gene contributes to a molecular signature distinguishing IBC from non-IBC samples (30). We aslo managed to identified several mutations in the primary lesion and numerous other gene mutations in the recurrent tissue, with the most significant being TP53. TP53, as a tumor suppressor gene, is commonly mutated in various tumors, including breast cancer, and patients with TP53 gene mutations are more susceptible to developing various malignant tumors (31, 32). In this study, we detected a nonsense mutation in exon 9 (p.Q331*) of the TP53 gene in the recurrent tissues, potentially leading to the inactivation of P53’s anti-tumor function, promoting tumor cell proliferation, migration, and anti-apoptosis, and contributing to tumor development. Moreover, it may result in resistance to chemotherapy drugs like platinum and fluorouracil. Several molecules targeting P53/TP53 have been developed, such as nutilins, MI-series analogs, PRIMA-1, and RITA, although only APR-246 and COTI-2 have progressed to clinical trials, while most others are still in the preclinical stage (33).

We also noted significantly higher abundances of PIK3CA and C4orf54 in the primary tumor compared to the skin recurrence tissues. PIK3CA mutations in breast cancer are diverse, with varying proportions in different breast cancer subtypes, being highest in HR+/HER2- disease, followed by HER2+ disease and TNBC (34). The FDA-approved α-selective PI3K inhibitor, Alpelisib, has demonstrated efficacy in treating patients with advanced PIK3CA-mutated HR+/HER2- breast cancer, based on positive results from the SOLAR-1 phase III randomized trial, which evaluated the combination of alpelisib and fulvestrant in multiple countries with PIK3CA-mutated, HR+/HER2- advanced breast cancer following progression on or after endocrine therapy (35). Moreover, although the frequency of PIK3CA mutations is low in HER2+ and TNBC, research interest in this pathway is growing, with ongoing studies exploring its significance in these two breast cancer subtypes (36, 37). As for C4orf54 (chromosome 4 open reading frame 54), it remains relatively understudied, yet it shows potential as a meaningful treatment target for breast cancer, underscoring the need for further research to better understand its role in breast cancer development and progression.

## Conclusion

The occurrence of bilateral inflammatory breast cancer, four years post-HER-2 positive breast cancer treatment, is exceptionally rare. We applied the standard rescue protocol for advanced HER-2 positive breast cancer. Our case underscores the vital role of consistent imaging and physical examinations in post-inflammatory breast cancer management, emphasizing patient compliance in battling this aggressive disease. Whole Exome Sequencing (WES) results provide promising insights for advancing breast cancer clinical target research. Our findings stress the need for ongoing research and targeted strategies to enhance the management and outcomes of this intricate condition.

## Data availability statement

Data supporting the findings of this study are available in the in the Genome Sequence Archive (Genomics, Proteomics & Bioinformatics 2021) in National Genomics Data Center (Nucleic Acids Res 2022), China National Center for Bioinformation / Beijing Institute of Genomics, Chinese Academy of Sciences (GSA-Human: HRA006283) that are publicly accessible at https://ngdc.cncb.ac.cn/gsa-human.

## Ethics statement

The studies involving humans were approved by Ethical Committee of Jiangsu University Affiliated People’ Hospital. The studies were conducted in accordance with the local legislation and institutional requirements. The participants provided their written informed consent to participate in this study. Written informed consent was obtained from the individual(s) for the publication of any potentially identifiable images or data included in this article.

## Author contributions

RQ: Writing – original draft. XW: Writing – original draft. TF: Data curation, Writing – review & editing. TW: Resources, Writing – review & editing. CL: Writing – review & editing. XS: Methodology, Writing – review & editing. LY: Writing – review & editing.

## Supplementary material

Supplementary Figure 1 KEGG analysis presented that both primary tumor (P) and skin recurrence (R) might be enriched in axon guidance signal pathways.

Supplementary Figure 2 Molecular function analysis showed that P and R may have changes in semaphorin receptor activity.

Supplementary Figure 3 Cellular component analysis indicated that the change of semaphorin receptor complex is detected in both P and R.
