# Supplementary figures and images for: Bilateral inflammatory recurrence of HER-2 positive breast cancer: a unique case report and literature review
Source: Front Oncol. 2024 Jan 12;14:1276637. doi: 10.3389/fonc.2024.1276637 (PMC10811202; doi:10.3389/fonc.2024.1276637)

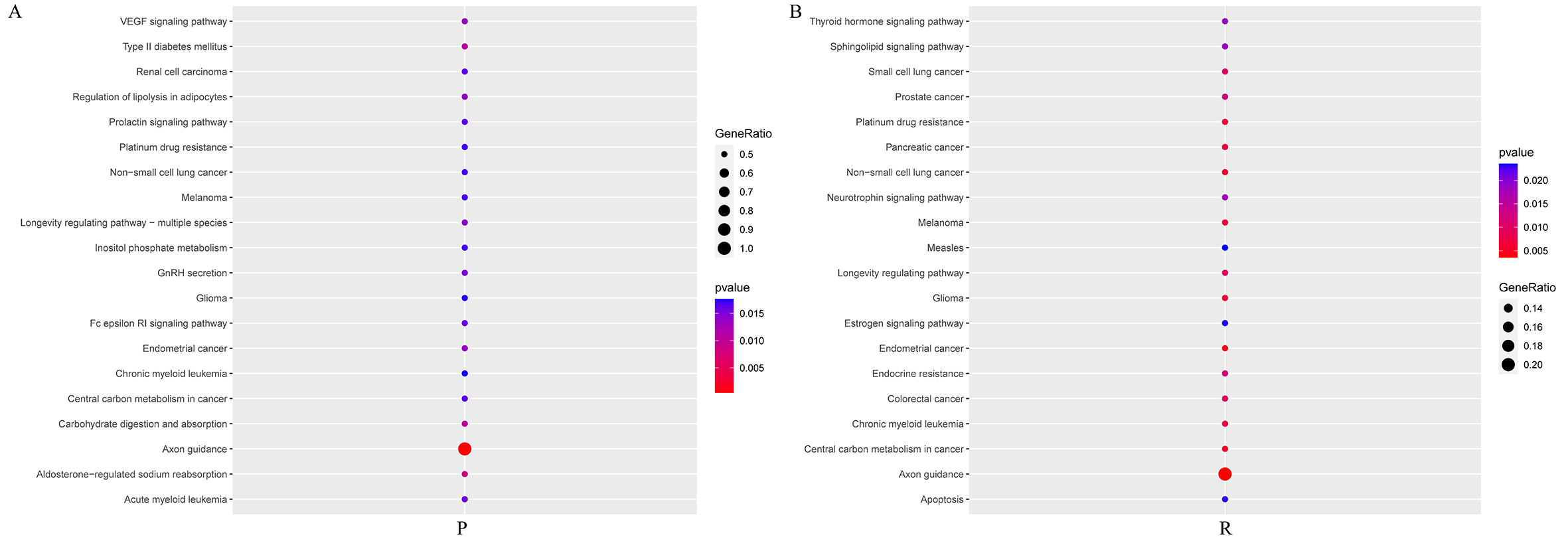

Supplement: Supplementary Figure 1 — KEGG analysis presented that both primary tumor (P) and skin recurrence (R) might be enriched in axon guidance signal pathways. [file Image_1.tif]

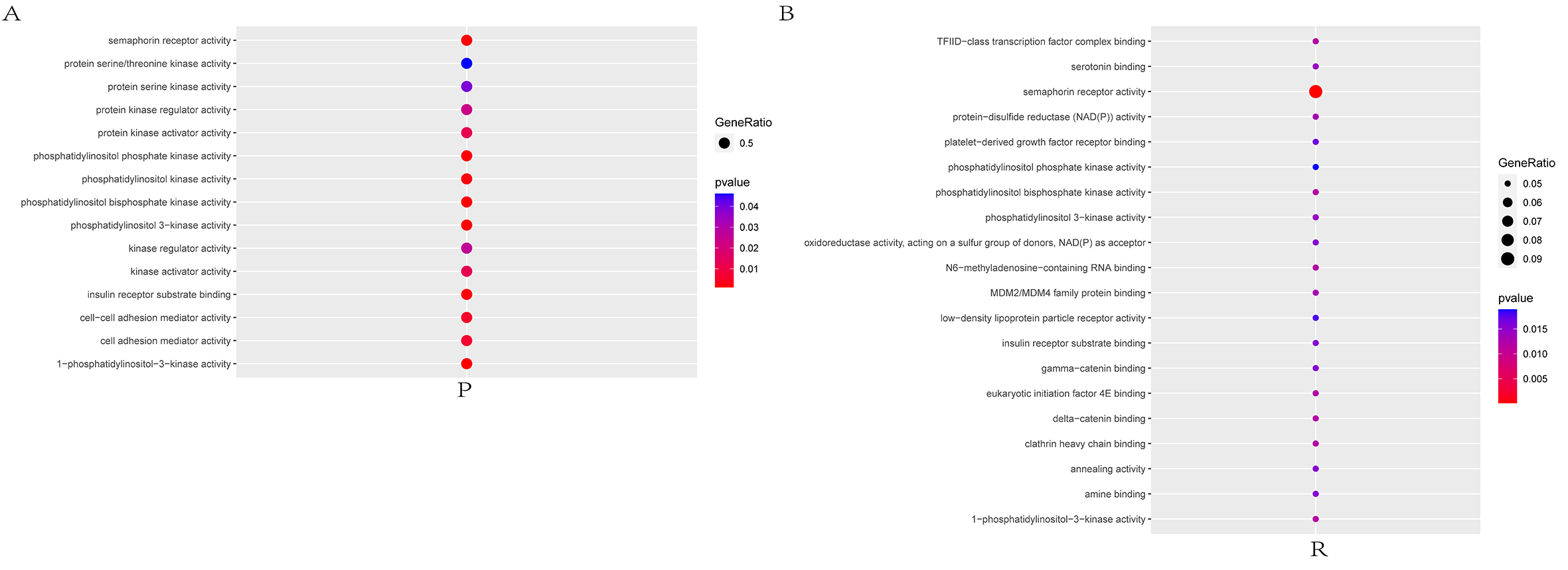

Supplement: Supplementary Figure 2 — Molecular function analysis showed that P and R may have changes in semaphorin receptor activity. [file Image_2.tif]

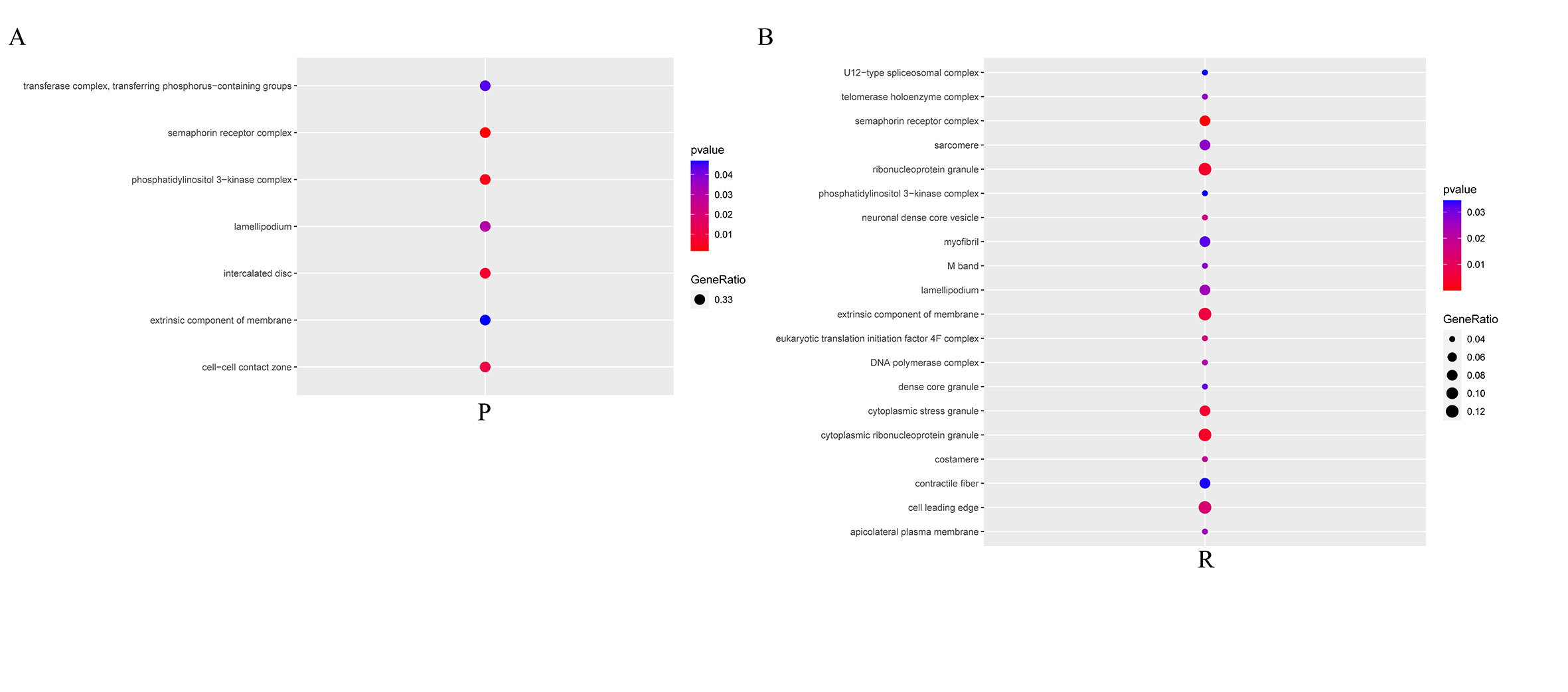

Supplement: Supplementary Figure 3 — Cellular component analysis indicated that the change of semaphorin receptor complex is detected in both P and R. [file Image_3.jpeg]
